# MBOAT7 rs641738 increases risk of liver inflammation and transition to fibrosis in chronic hepatitis C

**DOI:** 10.1038/ncomms12757

**Published:** 2016-09-15

**Authors:** Khaled Thabet, Anastasia Asimakopoulos, Maryam Shojaei, Manuel Romero-Gomez, Alessandra Mangia, William L. Irving, Thomas Berg, Gregory J. Dore, Henning Grønbæk, David Sheridan, Maria Lorena Abate, Elisabetta Bugianesi, Martin Weltman, Lindsay Mollison, Wendy Cheng, Stephen Riordan, Janett Fischer, Ulrich Spengler, Jacob Nattermann, Ahmed Wahid, Angela Rojas, Rose White, Mark W. Douglas, Duncan McLeod, Elizabeth Powell, Christopher Liddle, David van der Poorten, Jacob George, Mohammed Eslam, International Liver Disease Genetics Consortium, Rocio Gallego-Duran, Tanya Applegate, Margaret Bassendine, Chiara Rosso, Lavinia Mezzabotta, Reynold Leung, Barbara Malik, Gail Matthews, Jason Grebely, Vincenzo Fragomeli, Julie R. Jonsson, Rosanna Santaro

**Affiliations:** 1 Storr Liver Centre, Westmead Institute for Medical Research and Westmead Hospital, University of Sydney, Sydney, New South Wales 2145, Australia; 2 Department of Biochemistry, Faculty of Pharmacy, Minia University, Minia 6111, Egypt; 3 Centre for Immunology and Allergy Research, Westmead Institute for Medical Research, Sydney, New South Wales 2145, Australia; 4 Nepean Genomic Research Group, ICU, Nepean Hospital, Kingswood, New South Wales 2747, Australia; 5 Unit for The Clinical Management of Digestive Diseases and CIBERehd, Hospital Universitario de Valme, 41014 Sevilla, Spain; 6 Division of Hepatology, Ospedale Casa Sollievo della Sofferenza, IRCCS, 71013 San Giovanni Rotondo, Italy; 7 NIHR Biomedical Research Unit in Gastroenterology and the Liver, University of Nottingham, Nottingham NG7 2UH, UK; 8 Section of Hepatology, Clinic for Gastroenterology and Rheumatology, University Clinic Leipzig, 04103 Leipzig, Germany; 9 Kirby Institute, The University of New South Wales, Sydney, New South Wales 2052, Australia; 10 Department of Hepatology and Gastroenterology, Aarhus University Hospital, DK-8000 Aarhus, Denmark; 11 Institute of Translational and Stratified Medicine, Plymouth University, Plymouth PL4 8AA, UK; 12 Division of Gastroenterology and Hepatology, Department of Medical Science, University of Turin, 10126 Turin, Italy; 13 Department of Gastroenterology and Hepatology, Nepean Hospital, Sydney, New South Wales 2747, Australia; 14 Department of Gastroenterology and Hepatology, Fremantle Hospital, Fremantle, Western Australia 6160, Australia; 15 Department of Gastroenterology and Hepatology, Royal Perth Hospital, Western Australia 6000, Australia; 16 Gastrointestinal and Liver Unit, Prince of Wales Hospital and University of New South Wales, Sydney, New South Wales 2031, Australia; 17 Department of Internal Medicine I, University of Bonn, 53105 Bonn, Germany; 18 Centre for Infectious Diseases and Microbiology, Marie Bashir Institute for Infectious Diseases and Biosecurity, University of Sydney at Westmead Hospital, Westmead, New South Wales 2145, Australia; 19 Department of Anatomical Pathology, Institute of Clinical Pathology and Medical Research (ICPMR), Westmead Hospital, Sydney, New South Wales 2145, Australia; 20 Princess Alexandra Hospital, School of Medicine, The University of Queensland, Woolloongabba, Queensland 4102, Australia; 21 Institute of Translational Medicine, Newcastle University, Newcastle upon Tyne, UK

## Abstract

Cirrhosis likely shares common pathophysiological pathways despite arising from a variety of liver diseases. A recent GWAS identified rs641738, a polymorphism in the *MBOAT7* locus, as being associated with the development of alcoholic cirrhosis. Here we explore the role of this variant on liver inflammation and fibrosis in two cohorts of patients with chronic hepatitis C. In 2,051 patients, rs641738 associated with severe hepatic inflammation and increased risk of fibrosis, as well as fast fibrosis progression. At functional level, rs641738 associated with MBOAT7 transcript and protein levels in liver and blood, and with serum inflammatory, oxidative stress and macrophage activation markers. MBOAT7 was expressed in immune cell subsets, implying a role in hepatic inflammation. We conclude that the *MBOAT7* rs641738 polymorphism is a novel risk variant for liver inflammation in hepatitis C, and thereby for liver fibrosis.

## Abstract (web summary)

Chronic Hepatitis C infection is associated with a broad spectrum of liver pathologies, ranging from inflammation to fibrosis and liver cancer. Here Thabet *et al*. identified a polymorphism in the gene MBOAT7 that is associated with increased hepatic inflammation and higher risk of fibrosis development and progression.

## Graphical abstract

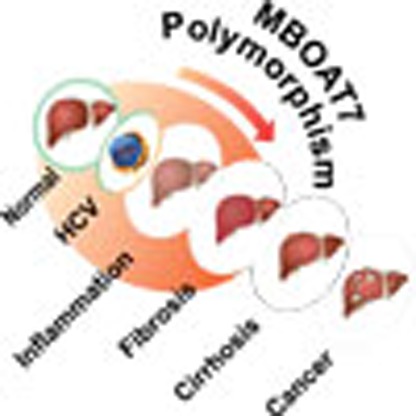

About 3% of the world population has been exposed to the hepatitis C virus (HCV), a leading cause of liver-related morbidity and mortality^1^. Not all patients with chronic hepatitis C (CHC) will develop serious sequelae, with prognosis and management largely depending on the extent and progression of liver inflammation and fibrosis^2^. Complex and as yet not fully understood interactions between the host, the virus and the environmental factors modulate these outcomes, with inflammation and fibrosis sharing common pathophysiological pathways, despite differences in the cause of liver injury^2^.

A recent genome-wide association study followed by fine mapping identified a novel single-nucleotide polymorphism (SNP; rs641738) in the membrane bound *O*-acyltransferase domain containing 7 gene (*MBOAT7*), also known as lysophosphatidylinositol acyltransferase 1 (*LPIAT1*), to be associated with alcoholic cirrhosis^3^. This association was recently extended to the full histological spectrum of non-alcoholic fatty liver disease^4^. MBOAT, a family of enzymes identified in 2000, comprises 11 genes that are involved in a variety of biological processes and are considered as candidate therapeutic targets for several diseases including obesity, viral infections, atherosclerosis and Alzheimer's disease^5^. MBOAT7 catalyses the transfer of an acyl-CoA to lysophosphatidylinositol (lysoPI) and has a preference for arachidonoyl-CoA. Thus, it might be one of the mechanisms by which pro-inflammatory free arachidonic acid and eicosanoid levels are regulated. Increased availability of arachidonic acid leads to inflammatory signalling by prostaglandins and leukotrienes, potent chemoattractant mediators of inflammation^6^.

We tested the hypothesis that *MBOAT7* is a liver injury risk locus in two large, well-characterized cohort of patients with CHC, some of whom have been the subject of previous reports^7^,^8^. Here we demonstrate that *MBOAT7* rs641738 associates with hepatic inflammation and the risk of liver fibrosis, as well as fibrosis progression rate (FPR). MBOAT7 rs641738 regulated MBOAT7 expression in opposite directions in liver and blood, and associated with serum inflammatory, oxidative stress and macrophage activation markers. MBOAT7 was expressed in immune cell subsets, implying a role in hepatic inflammation. These findings suggest that MBOAT7 is a novel liver injury risk locus in CHC.

## Results

### Patient characteristics

Baseline characteristics of the discovery, validation and overall cohorts of patients are shown in Supplementary Table 1. The median age was 45 years with 65% being male. About half (55%) had significant fibrosis (Metavir score F2–4) and 45% had moderate/severe necroinflammation (Metavir A2–A3). The discovery cohort included a higher percentage of males who were younger at time of biopsy, with higher liver enzymes. Both cohorts were similar with respect to body mass index (BMI), platelet count, HCV-RNA levels and the distribution of daily alcohol consumption over 50 g. The prevalence of significant fibrosis was similar between the two cohorts. The discovery cohort had more HCV genotype 3, and the validation cohort more HCV genotype 1.

### Genotype distribution, HWE calculations and genetic model

The genotype distribution of *MBOAT7* rs641738 was in Hardy–Weinberg equilibrium (*P*=0.1 for both CHC and healthy controls cohorts). The minor allele frequency (MAF) was 0.45, similar to that of the 270 Caucasian self-reported ‘healthy' controls (MAF=0.45) and consistent with that previously reported in the genome-wide association study on alcoholic cirrhosis^3^, and in the healthy Caucasian population by the 1000 genome project (Supplementary Table 2). There was no significant difference in rs641738 allele frequency distribution according to patient country of origin (Australia, UK, Germany, Italy and Spain; *P*=0.8 for trend by the Cochran–Armitage test).

Owing to a lack of current knowledge regarding the genetic model of inheritance that might explain the effect of this variant, we avoided choosing an *a priori* or arbitrary model as recommended^9^.The best model is defined as the one with the smallest Akaike information criterion (AIC) value. The dominant model for the minor allele (T) best fitted the data and was the most appropriate, as it had the lowest AIC value (Supplementary Table 3). Hence, rs641738 comparisons were made using a dominant model for the minor allele (T), unless otherwise indicated.

### Viral and clinical characteristics stratified by rs641738 genotype

Apart from the fact that subjects with the rs641738 CC genotype had significantly lower total leukocyte levels compared with subjects with CT and TT, no other significant associations were observed (Supplementary Table 4) with clinical characteristics, liver enzymes, lipid profile or HCV-RNA. An association with the leukocyte level was also significant when comparing the different genotypes (*P*=0.04, by analysis of variance after Bonferroni correction for multiple comparisons).

To fully test the role of rs641738 in CHC, several primary and exploratory analyses were undertaken. Primary analyses aimed to test the association of rs641738 with liver injury (that is, hepatic inflammation, fibrosis and fibrosis progression). The exploratory analyses tested for the association of rs641738 with hepatic steatosis and hepatocellular carcinoma (HCC).

### rs641738 and hepatic inflammation and fibrosis in CHC

**Figure 1:**
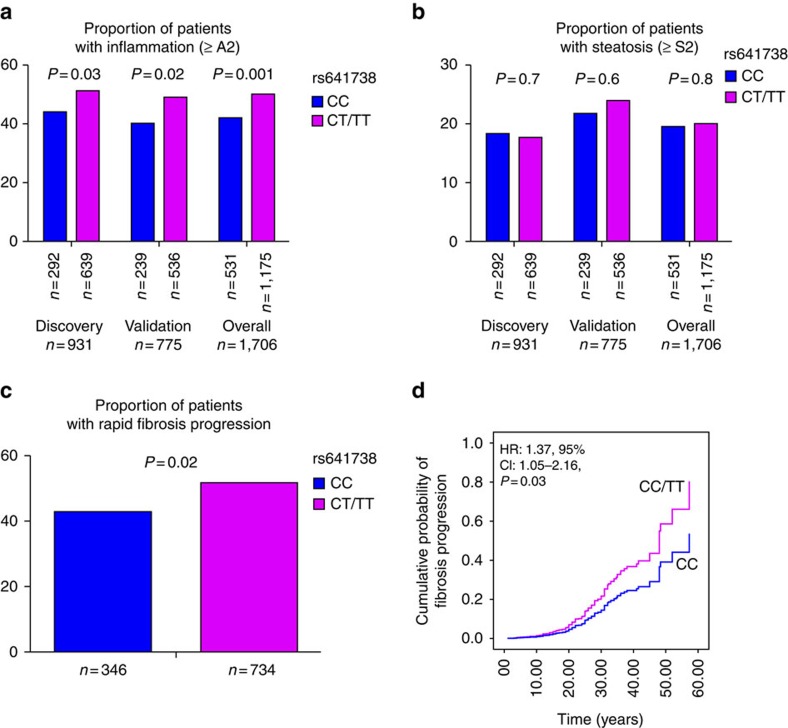
Association of rs641738 genotype with liver injury. Association of rs641738 genotype with necroinflammation (**a**) and steatosis degree (**b**) by discovery (*n*=931), validation (*n*=775) and overall cohorts (*n*=1706). *P* values are univariate and provided for the dominant model of inheritance. Association of rs641738 genotype with FPR in the sub-cohort with an estimated date of infection (*n*=1,080) (**c**) with fast fibrosis progression. (**d**) Multivariate Cox regression analysis of rs641738 genotypes on the cumulative probability of progression to any fibrosis (≥F1), adjusted for age, gender, BMI, *PNPLA3* rs738409, *TM6SF2* rs58542926 and *IFNL* rs12979860 genotype. HR, hazard ratio.

Necroinflammatory activity was more pronounced in patients carrying the minor allele (T) of MBOAT7 than in patients carrying the major genotype, that is, a dominant model for the minor allele. In the discovery cohort, the proportion of patients with an activity score of ≥2 was significantly higher in CT/TT patients as compared with CC patients (51% versus 44%, *P*=0.03); this was replicated in the validation cohort (49% versus 40%, *P*=0.02) and the overall cohort (50% versus 42%, *P*=0.001; Fig. 1a). By multiple logistic regression analysis adjusted for age, gender, BMI, homeostatic model assessment of insulin resistance (HOMA-IR), recruitment centre, alcohol consumption, viral load, *PNPLA3* rs738409, *TM6SF2* rs58542926 and *IFNL* rs12979860 genotype, rs641738 CT/TT was independently associated with higher necroinflammatory activity (odds ratio (OR): 1.44; 95% confidence interval (CI): 1.14–1.72; *P*=0.001; Supplementary Table 5).

Using a recessive mode of inheritance, the proportion of patients with any fibrosis (≥F1) was significantly higher in TT compared with CC/CT patients (89% versus 82%, *P*=0.04). The same trend was observed in the validation cohort (94% versus 89%, *P*=0.05) and was more significant in the overall cohort (91% versus 86%, *P*=0.006). This finding remained significant in multiple logistic regression analyses adjusted for the same variables listed above (OR: 1.69; 95% CI: 1.11–3.57; *P*=0.02; Supplementary Table 5). Notably, rs641738 did not influence the likelihood of severe fibrosis (≥F3 versus F0–F2) (OR: 1.03; 95% CI: 0.80–1.34; *P*=0.7), significant fibrosis (≥F2 versus F0/F1) or cirrhosis (F4 versus F0–F3), including when adopting other genetic models. These data suggest that the effect of rs641738 is restricted to the transition to fibrosis from an absence of fibrosis (F0).

To further confirm this observation, we undertook analysis in the sub-cohort with absent or mild fibrosis (*n*=776) and compared the effect of rs641738 on F0 versus F1 only. The analysis was restricted to the overall cohort to ensure adequate power. Again, the TT genotype was associated with an increased risk for the transition to fibrosis (OR: 1.78; 95% CI: 1.2–2.64; *P*=0.004), a finding that remained significant in multiple logistic regression analysis (OR: 1.69; 95% CI: 1.12–2.54; *P*=0.01).

For additional confirmation, we undertook analysis in the 1,080 patients with CHC and a known duration of infection, allowing us to assess the relationship with fibrosis progression. The baseline characteristics of the cohort were similar among subjects included and not included in the fibrosis progression sub-analysis (Supplementary Table 6). The proportion of patients with fast FPR was significantly higher in CT/TT patients as compared with CC patients (52% versus 43%, *P*=0.02; Fig. 1c). The adjusted OR of the rs641738 (T) allele for having fast FPR was 1.56; 95% CI: 1.03–2.36; *P*=0.03. Because, fibrosis progression may not be constant over time and FPR assumes linearity, we undertook further complementary analysis using multivariate Cox proportional hazards adjusted for age, gender, BMI, *PNPLA3* rs738409, *TM6SF2* rs58542926 and *IFNL* rs12979860 genotype. Again, the rs641738 (T) allele was associated with an increase in the hazard of progression to fibrosis (≥F1; hazard ratio: 1.37; 95% CI: 1.05–2.16; *P*=0.03; Fig. 1d).

### MBOAT7 rs641738 and hepatic steatosis

Patients carrying the *MBOAT7* rs641738 (T) allele had a similar distribution of steatosis grade as those with CC genotype (*P*=0.8, Fig. 1b). In univariate and multivariate analyses adjusted for the same variables, rs641738 (T) allele was not associated with either the severity of steatosis when subdividing the cohort according to absent/mild steatosis (S0–S1) compared with moderate/severe steatosis (S2–S3; OR: 0.99; 95% CI: 0.74–1.33; *P*=0.9; Supplementary Table 5) or with the presence of steatosis (S0 versus S1–S3; OR: 0.92; 95% CI: 0.0.72–1.17; *P*=0.5). No difference was observed when stratifying the cohort according to HCV genotype (genotype 3 (*n*=343) versus non-3 (*n*=1363)) based on the direct effect of genotype 3 on steatosis^10^, or by adopting other genetic models, that is, additive or recessive models.

Notably, there was no interaction observed between *MBOAT7* rs641738 and either *PNPLA3* rs738409 or *TM6SF2* rs58542926 genotype regarding any of the histological features. Thus, the effect of these three loci seems to be independent of each other, suggesting that they regulate different pathways.

In sum, these data suggest that the *MBOAT7* rs641738 (T) allele has a more profound effect on hepatic inflammation and the transition from absent fibrosis to early-stage liver fibrosis (F1), with no effect on fibrosis progression to later stages (F2–4). This effect was not due to an influence on hepatic steatosis and independent of *PNPLA3* rs738409, *TM6SF2* rs58542926 and *IFNL* rs12979860 genotype.

### MBOAT7 rs641738 genotype and HCC

HCC is an inflammation-induced cancer^11^; hence, we determined whether *MBOAT7* rs641738 had an effect on HCV-related HCC. The *MBOAT7* rs641738 allele and genotype frequencies were compared in 75 Caucasian patients with HCV-related HCC to the entire CHC cohort described above (*n*=1,706; Supplementary Table 7). rs641738 MAF in the HCC cohort was 0.44, similar to the CHC cohort; no significant association was observed with HCC (OR: 0.96; 95% CI: 0.58–1.57; *P*=0.8) in multivariate analysis after incorporating known risk factors, including age, gender, BMI and Child-Pugh score. Again, these findings imply that the role of rs641738 is limited to the early stages of liver disease, but not to further progression or occurrence of HCC.

### rs641738 associates with MBOAT7 expression in liver and blood

**Figure 2:**
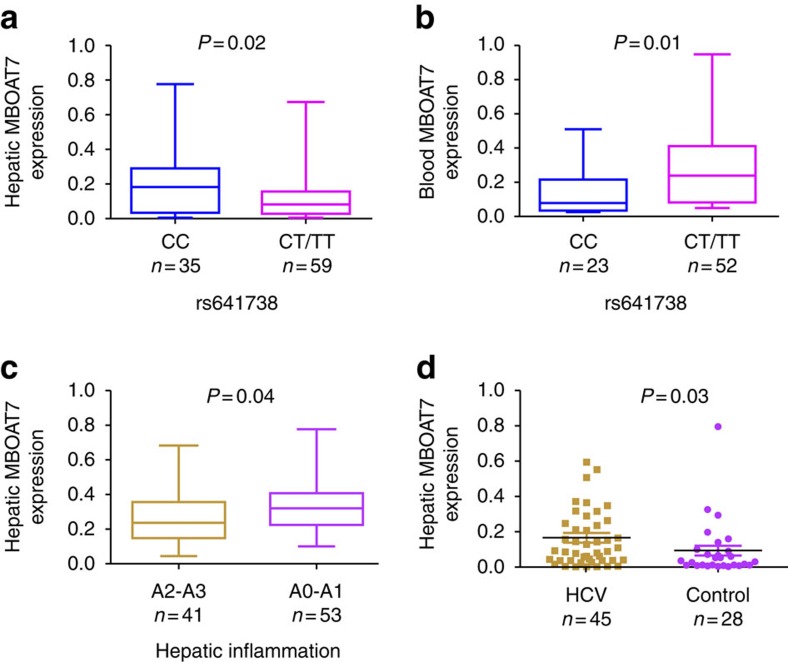
Association of rs641738 genotype with MBOAT7 mRNA expression. Association between *MBOAT7* rs641738 genotype and hepatic (*n*=94) (**a**) and blood (*n*=75) (**b**) MBOAT7 mRNA levels. Clinical and anthropometric characteristics of these patients are shown in Supplementary Table 8. The *x* axis shows the genotypes at rs641738 using the dominant model of inheritance (CC=35 and CT/TT=59 in the liver biopsy cohort, and CC=23 and CT/TT=52 in the blood cohort) and the *y* axis shows the MBOAT7 expression level relative to GAPDH by quantitative real-time PCR. (**c**) Hepatic MBOAT7 mRNA levels according to hepatic inflammation. The *x* axis shows hepatic inflammation dichotomized as absent/mild (METAVIR score A0–A1) (*n*=53) or moderate/severe (METAVIR score A2–A3) (*n*=41), and the *y* axis shows hepatic MBOAT7 expression as fold change. The number of independent samples tested in each group is shown in parentheses. Each group is shown as a box plot and the median values are shown as thick dark horizontal lines. The box covers the twenty-fifth to seventy-fifth percentiles. We tested the difference in the median values among genotypes using the two-tailed Mann–Whitney tests. We plotted the box plots using Graphpad prism 5. (**d**) Comparison of hepatic MBOAT7 mRNA levels in hepatitis C patients with no or mild fibrosis (F0–F1) (*n*=45) and control (*n*=28). The number of independent samples tested in each group is shown in parentheses. Median and interquartile range are shown and *P* value was calculated using the two-tailed Mann–Whitney test.

The association of rs641738 genotypes with the hepatic expression of MBOAT7 mRNA and protein in patients with CHC is unknown. In 94 liver biopsies of a sub-cohort of patients whose baseline characteristics matched that of the overall cohort (summarized in Supplementary Table 8), there was a significant relationship between rs641738 genotype and MBOAT7 mRNA expression (*P*=0.03, Supplementary Fig. 1A); the same was observed in analysis comparing subjects carrying the CC genotype versus the CT/TT genotype (*P*=0.02; Fig. 2a). The rs641738 (T) allele remained independently associated with low hepatic MBOAT7 mRNA expression after adjustment for age, sex, BMI, alanine aminotransferase (ALT) and severity of liver disease (estimate −0.095±0.02; *P*=0.03). In a further analysis, MBOAT7 expression was significantly higher in subjects with none or mild hepatic inflammation (A0–A1), compared with those with significant hepatic inflammation (A2–A3; Fig. 2c). To determine if MBOAT7 expression is an adaptive response consequent to liver injury, we compared MBOAT7 expression in CHC with low fibrosis (F0–F1) (*n*=45) with 28 controls without liver disease. MBOAT7 demonstrated significantly higher expression in CHC (*P*<0.01; Fig. 2d). This remained significant after stratification by MBOAT7 genotype (*P*<0.05 for both alleles).

**Figure 3:**
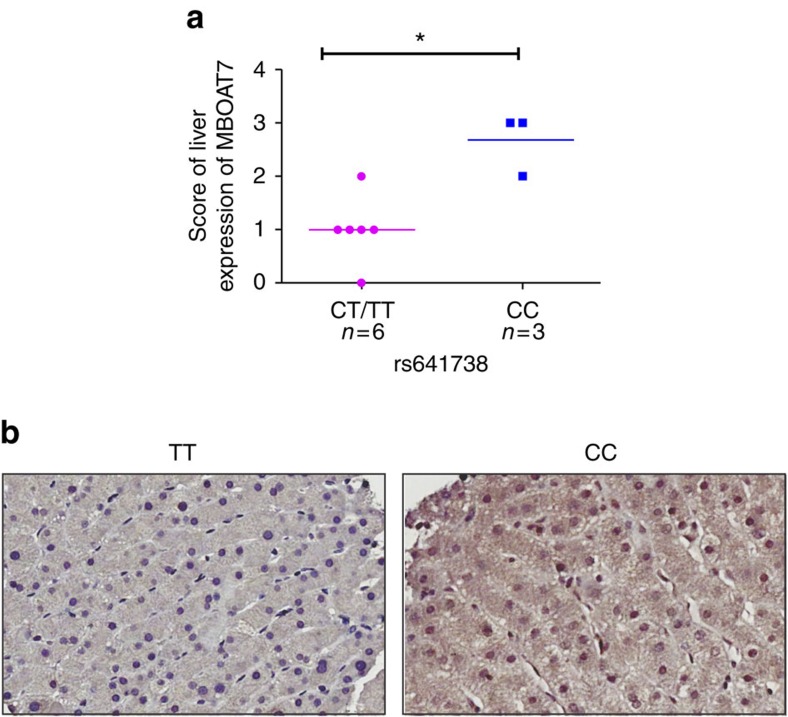
Association of rs641738 genotype with MBOAT7 protein expression. Association between hepatic *MBOAT7* rs641738 genotype and MBOAT7 protein expression assessed using immunohistochemistry (IHC). (**a**) Scores for liver protein expression of MBOAT7 was evaluated using IHC according to grouping of rs641738 genotype using the dominant model of inheritance. The *x* axis shows the genotypes at rs641738 using the dominant model of inheritance (CC=3 and CT/TT=6) and the *y* axis shows the score of liver protein expression of MBOAT7. The number of independent samples tested in each group is shown in parentheses. (**b**) Representative liver expression pattern of MBOAT7 in patients carrying the rs641738 CC or TT genotype, respectively. MBOAT7 immunoreactivity was examined using light microscopy of liver sections. Original magnification × 400.

For further confirmation, we explored the level of MBOAT7 protein expression according to rs641738 genotype by immunohistochemistry using specific human anti-MBOAT7 antibody (*n*=9 subjects). Owing to the correlation between MBOAT7 and hepatic inflammation and fibrosis, and to minimize confounding, we restricted analysis to only subjects with no (F0) or early fibrosis (F1). We observed that MBOAT7 immunoreactivity was mainly in hepatocytes with predominant cytoplasmic staining. We demonstrated significantly reduced MBOAT7 immunoreactivity in subjects carrying the (T) allele, compared with those with CC genotype (*P*<0.03; Fig. 3).

We then explored whether *MBOAT7* rs641738 regulates transcript expression in blood as recent data indicates that expression quantitative trait loci (eQTLs) may affect gene expression in a tissue- and cell-dependent manner^12^. Interestingly, rs641738 regulated MBOAT7 transcript expression in blood (*n*=75) in an opposite direction to that in liver, with the (T) allele having the highest expression (Fig. 2 and Supplementary Fig. 1B).

To identify the functional downstream effects of rs641738, we conducted bioinformatics analysis. This demonstrated that the rs641738 SNP is located in potentially functional regions containing predicted transcription factor-binding motifs with protein-binding sequences as demonstrated by ChIP-seq, located in the chromatin structures and the histone modification regions. The RegulomeDB score was 1b indicating that the SNP is likely to be functional (Supplementary Table 9) (RegulomeDB score is scaled from 1a to 6 and the lower the score the more likely that the SNP affects binding and is linked to the expression of a gene target; see Supplementary Methods for more details). rs641738 was not associated with microRNA-predicted binding affinity changes.

### MBOAT7 is expressed by immune cells

**Figure 4:**
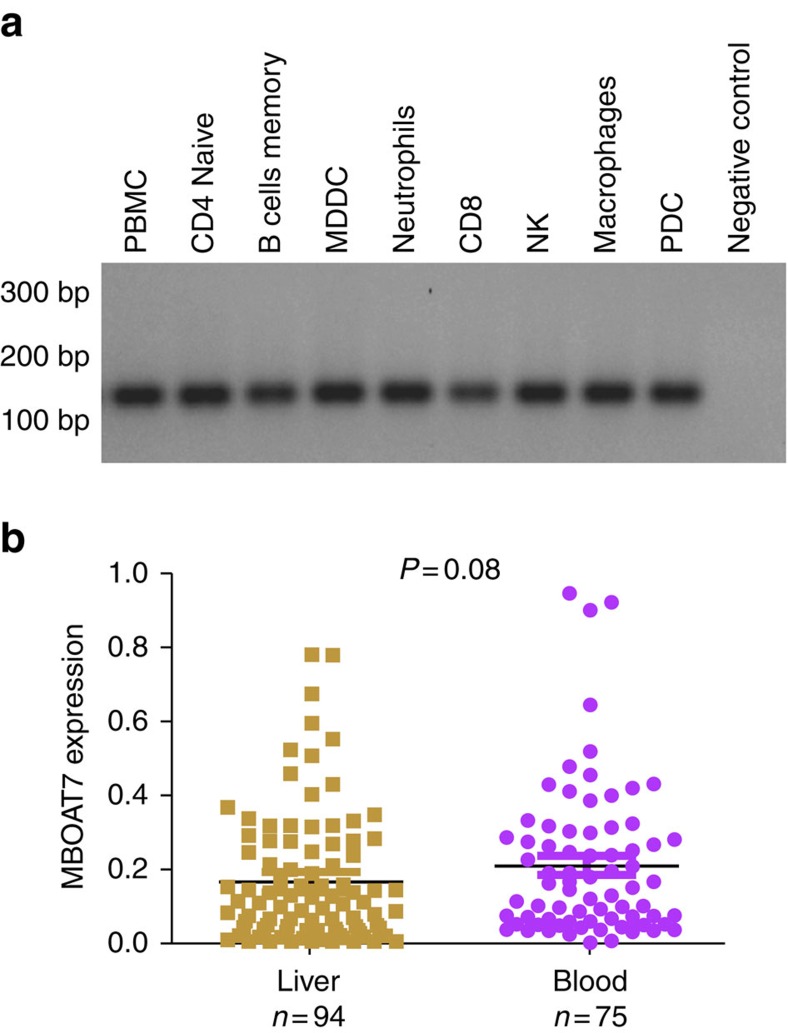
Expression of MBOAT7 in immune cells. (**a**) Expression of MBOAT7 on circulating immune cells (PBMCs, T and B lymphocytes, monocytes, macrophages, natural killer cells, dendritic cells (plasmacytoid dendritic cells (PDCs) and monocyte-derived dendritic cells (MDDC)) and neutrophils). (**b**) Comparison of the expression of MBOAT7 in blood (*n*=75) and liver (*n*=94) in patients with CHC. The number of independent samples tested in each group is shown in parentheses. Median and interquartile range are shown; *P* value was calculated using the two-tailed Mann–Whitney test.

HCV associated liver pathology is characterized by an inflammatory infiltrate consisting of different immune cell populations. The magnitude of this inflammatory reaction determines histologic inflammatory activity reflected in the METAVIR score, and is one of the major predictors of progressive liver damage^13^. Apart from neutrophils^14^, MBOAT7 expression on other immune cells has not been investigated. We therefore examined the expression of MBOAT7 in other immune cell subsets (peripheral blood mononuclear cells (PBMCs), T and B lymphocytes, monocytes, macrophages, natural killer cells, dendritic cells and neutrophils); all cell types demonstrated good expression of MBOAT7 (Fig. 4a). Further, MBOAT7 expression in whole blood from 75 CHC patients was comparable to that in HCV-infected liver biopsies (Fig. 4b).

### rs641738 genotype and markers of inflammation

**Figure 5:**
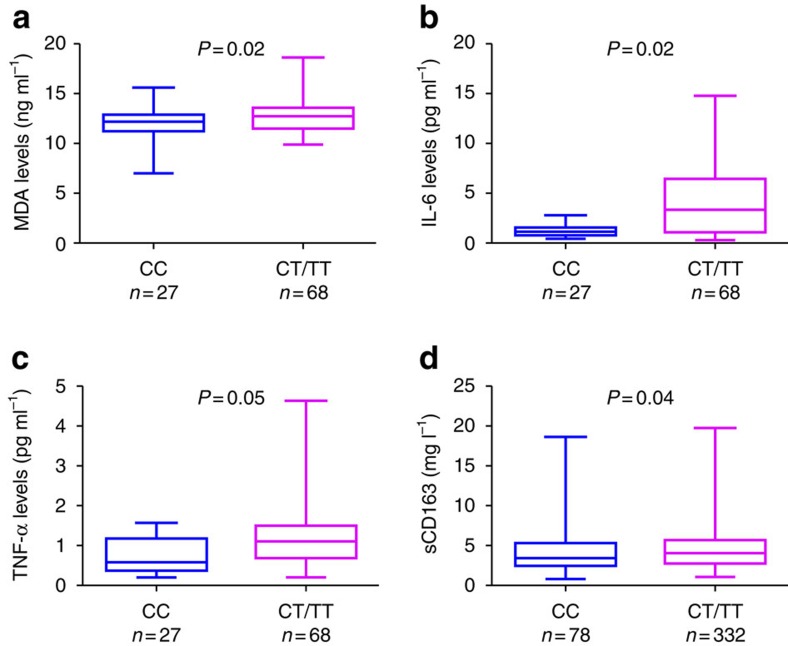
Association between rs641738 genotype and inflammatory, oxidative stress and macrophage activation markers. *rs641738* genotype correlation with MDA, an oxidative stress marker (**a**), IL-6 (**b**) and TNF-α, as inflammatory markers (**c**). Clinical and anthropometric characteristics of these patients are shown in Supplementary Table 8 (*n*=95). The *x* axis shows the genotypes at rs641738 using the dominant model of inheritance (CC=27 and CT/TT=68) and the *y* axis shows serum MDA as ng ml^−1^ (**a**), IL-6 as pg ml^−1^, TNF-α as pg ml^−1^. (**d**) Association between *MBOAT7* rs641738 genotype and the macrophage activation marker sCD163 (*n*=510). The *x* axis shows the genotypes at rs641738 using the dominant model of inheritance (CC=178 and CT/TT=332) and the *y* axis shows sCD163 as mg ml^−1^. The number of independent samples tested in each group is shown in parentheses. Each group is shown as a box plot and the median values are shown as thick dark horizontal lines. The box covers the twenty-fifth to seventy-fifth percentiles. We tested the difference in the median values among genotypes using the two-tailed Mann–Whitney test. We plotted the box plots using Graphpad prism 5.

To characterize the potential role of rs641738, we explored its association with serum inflammatory and oxidative stress markers (tumour necrosis factor-α (TNF-α), interleukin-6 (IL-6) and malondialdehyde (MDA)) in the sub-cohort of 95 subjects. Their clinical and anthropometric characteristics are shown in Supplementary Table 8. Consistent with the genetic data, TNF-α levels, IL-6 levels and oxidative stress as measured by MDA were higher for subjects carrying the rs641738 (T) allele compared with those with the CC genotype (*P*<0.05, for all; Fig. 5).

We next examined the expression of other proinflammatory cytokines to determine whether they differed by rs641738 genotype. To do this, we quantified the mRNA expression of TNF-α, IL-1b, IL-6, CCL-19 and CCL-21 in blood from 75 patients. As shown in Supplementary Fig. 2, as well as having higher TNF-α and IL-6 expression, subjects with the minor (T) allele also had higher IL-1b compared with those with the major CC genotype.

### rs641738 and macrophage activation markers

Owing to the high expression of MBOAT7 on immune cells, we also explored the correlation of *MBOAT7* rs641738 with serum sCD163, a macrophage activation marker. Interestingly, subjects with rs641738 (T) allele had significantly higher serum sCD163 compared with those with the CC genotype (*P*=0.04; Fig. 5).We recently reported that sCD163 is associated with the severity of liver disease^15^. To exclude the possibility that the association was secondary to worsening liver disease, in a multivariable regression model adjusting for age, the severity of liver disease and leukocyte count, rs641738 remained independently associated with serum sCD163 (*P*=0.032).

## Discussion

We evaluated the role of *MBOAT7* rs641738, recently reported to be a risk variant for alcoholic cirrhosis^3^, in two independent large cohorts of patients with CHC. We observed that (1) *MBOAT7* rs641738 is independently associated with inflammation and the transition to early fibrosis; (2) hepatic MBOAT7 expression correlates with inflammation; (3) rs641738 genotype has no association with the severity of hepatic steatosis, HCC occurrence; (4) rs641738 genotype associates with MBOAT7 transcript and protein expression in liver and blood in opposite directions, and with serum inflammatory, oxidative stress and macrophage activation markers; and (5) MBOAT7 is well expressed by all major immune cell subsets.

MBOAT7 (LPIAT1) is involved in membrane phospholipid remodelling as it catalyses the transfer of an acyl-CoA to lysophosphatidylinositol (lysoPI) with a high preference for arachidonoyl-CoA. Thus, increased free arachidonic acid levels in the context of reduced MBOAT7 expression may lead to inflammatory signalling by arachidonic acid-derived prostaglandins and leukotrienes^14^. On the other hand, PI accumulation, perhaps by reducing arachidonic acid levels, has been identified as a potent anti-inflammatory molecule^16^.

In two cohorts of CHC patients comprising over 1,700 patients, *MBOAT7* rs641738 (T) allele associates with hepatic inflammation and the transition from normal liver to early fibrosis and rapid fibrosis progression. Notably, the effect of this variant disappeared with progression of disease, as it had no effect on the late stages of fibrosis, cirrhosis or the occurrence of HCC. The influence of rs641738 on inflammation appears to be more profound than on fibrosis, as inflammation risk is increased in carriers of even one copy of the risk rs641738 (T) allele. On the other hand, for fibrosis, two copies of the risk allele are required to have the effect, which was mainly for the transition from F0 to F1 fibrosis. The predominant association with inflammation was corroborated by coordinate changes in serum inflammatory, oxidative stress and macrophage activation markers (TNF-α, IL-6, MDA and sCD163). Collectively, these results imply that MBOAT7 in the context of high expression plays an anti-inflammatory role and thus may be a compensatory adaptation during the early stages of HCV-induced inflammation. However, in the context of ongoing liver injury, this homeostatic response might possibly be no longer able to limit inflammation and fibrosis. This hypothesis is supported by the observed negative correlation between hepatic MBOAT7 expression and the extent of liver inflammation and the elevated hepatic MBOAT7 expression in CHC patients with no or mild fibrosis, compared with controls.

To provide contextual insights on the potential mechanisms by which MBOAT7 might influence inflammation and fibrosis, we considered several possibilities. MBOAT7 is involved in lipid processing that has been linked to both the development of liver inflammation and fibrosis^17^,^18^. However, we did not observe any association of rs641738 with steatosis. Thus, the association with inflammation and fibrosis is likely independent of lipid accumulation and insulin resistance, and also of any other lipid-related variants such as *PNPLA3* and *TM6SF2*.

Activation of innate immune pathways in hepatocytes following HCV infection leads to infiltration by inflammatory immune cell populations^18^. The magnitude of this response is one of the major predictors of progressive liver damage^19^. We demonstrated strong MBOAT7 expression on all immune cell subsets that infiltrate the liver during viral hepatitis. Further, expression in these subsets was overall comparable to that in HCV-infected liver biopsies, suggesting that it is part of a broad adaptive response to injury.

Our expression analysis of MBOAT7 in peripheral blood revealed an intriguing dissimilarity to liver, with the pro-inflammatory risk allele (T) having higher expression in blood but lower expression in liver. This result is consistent with eQTL analysis of publicly available databases for rs641738 (ref. 20) and recent data on non-alcoholic fatty liver disease^4^. These findings, in line with several reports on other SNPs, suggest that many genetic polymorphisms exert opposite effects on gene expression in different cell types and tissue contexts^21^. This could be explained by varying context-dependent regulatory mechanisms for gene expression. However, an alternative explanation may be that with initiation of liver injury, MBOAT7-expressing immune cell recruitment to liver occurs in a genotype dependent manner and is less in subjects with the risk T allele. Of relevance, those with the T allele have significantly higher leukocytes compared with those with the CC genotype. However, further study of MBOAT7-expressing immune cell trafficking in response to injury is required, before this hypothesis can be proven. Notably, rs641738 is located in potentially functional regions that contain predicted transcription factor-binding motifs.

In conclusion, we provide evidence for a role of *MBOAT7* rs641738 as a novel risk variant for hepatic inflammation and fibrosis in CHC. As MBOAT7 is expressed by all the principal immune cell subsets, it could be considered as a potential target to limit tissue inflammation in a variety of contexts.

## Methods

### Patient cohort

The study comprised 2,051 Caucasian consecutive subjects, including 1,706 with CHC, 931 in the discovery cohort and 775 in the validation cohort; 270 healthy controls and 75 with HCV-related HCC. Both the discovery and validation cohorts are from the International Liver Disease Genetics Consortium (ILDGC) database. Details of the cohort and inclusion criteria have been reported^7^,^8^. Briefly, all consecutive subjects with HCV-RNA serum positivity of Caucasian descent who had (a) a liver biopsy with scoring for fibrosis stage and disease activity before anti-viral treatment, (b) genomic DNA and (c) provided written consent for genetic analyses were included. Patients were excluded if they had evidence of other liver diseases by standard tests including co-infection with hepatitis B (surface antigen positivity) or human immunodeficiency virus, Wilson's disease, hereditary haemochromatosis, alpha1-antitrypsin deficiency or autoimmune hepatitis.

### Methods to estimate the duration of infection

Fibrosis progression was examined in 1,080 CHC patients with a reliable estimated duration of infection^7^,^22^. For subjects with a history of injecting drug use, the time of infection was estimated using the reported ‘first year of injection'. For patients with a history of blood transfusion, the onset of infection was assumed to be the year of transfusion. For patients with a history of occupational exposure, the onset of infection was assumed to be the first year of needle stick exposure. The duration of infection was calculated by subtracting the estimated age at infection from age at biopsy. FPR was determined by calculating the ratio between the fibrosis stage and the estimated disease duration (in years).

The healthy Caucasian European control group was enrolled from Westmead hospital, Sydney. They reported no history of chronic liver disease did not abuse alcohol (<20 gm of alcohol daily) and did not have any of the features of the metabolic syndrome according to the National Cholesterol Education Program Adult Treatment Panel III criteria.

Ethics approvals were obtained from the Human Research Ethics Committees of the Sydney West Area Health Service and the University of Sydney; Human Research Ethics Committee; South Eastern Sydney Local Health District, NSW Health; Ethic committee of St Vincent's Hospital, Sydney; The Royal Perth Hospital Human Research Ethics Committee, WA; Metro South Human Research Ethics Committee, QLD; Human Research Ethics Committee, South Metropolitan Health Service, Fremantle, WA; Sir Charles Gairdner Hospital Human Research Ethics Committee, WA; Ethic Committee of Valme University Hospital, Seville; Ethics Committees of Medical Research of the University of Leipzig, Berlin; Ethics Committees of Medical Research the University of Berlin (Charité), Berlin; Northern & Yorkshire MREC, Nottingham; Ethical Committee of the Città della Salute e della Scienza University Hospital, Torino; Ethical Committee IRCCS ‘Casa Sollievo della Sofferenza' Hospital San Giovanni Rotondo; Northumberland Research Ethics Committee, Newcastle; Ethics Committee of the Medical Faculty, Rheinische Friedrich-Wilhelms University Bonn, Bonn. All patients provided written informed consent.

### Clinical and laboratory assessment

The following data were collected at time of liver biopsy for all patients: gender; age; ethnicity; recruitment centre, alcohol intake (g per day), BMI and routine laboratory tests. Alcohol consumption was assessed by two separate interviews with the patient and close family members. BMI was calculated as weight divided by the square of the height (kg m^−2^). The homeostasis model assessment (HOMA-IR) was calculated as (fasting serum insulin (μU ml^−1^) × fasting serum glucose (mmol l^−^))/22.5.

### Genotyping

Genotyping for *MBOAT7* rs641738 was undertaken using the TaqMan SNP genotyping allelic discrimination method (Applied Biosystems, Foster City, CA, USA). All genotyping was blinded to clinical variables. *PNPLA3* rs738409, *TM6SF2* rs58542926 and *IFNL* rs12979860 genotyping were extracted from our previous reports^7^,^8^.

### Liver histopathology

Liver histopathology was scored according to METAVIR^23^. Fibrosis was staged from F0 (no fibrosis) to F4 (cirrhosis). Necroinflammation (A) was graded as A0 (absent), A1 (mild), A2 (moderate) or A3 (severe). The inter-observer agreement between pathologists was studied previously and was good (*κ*=77.5) for METAVIR staging using *κ* statistics^24^. We further tested for the inter-observer agreement between pathologists for fibrosis stage (F0–F1) and it was excellent (*κ*=90). All pathology assessment was blinded to genotyping results.

### Determination of inflammatory and oxidative stress markers

Serum TNF-α and IL-6 were measured by sandwich enzyme-linked immunosorbent assay (ELISA) and MDA was measured by reverse phase high performance liquid chromatography in 95 subjects^25^,^26^.

### Determination of macrophage activation markers

The plasma concentration of sCD163 was determined in duplicate in samples that were collected at the time of liver biopsy and stored at −80 °C by an in-house sandwich ELISA using a BEP-2000 ELISA-analyser (Dade Behring) in 510 patients^27^. Soluble CD163 is resistant to repeated freezing and thawing^27^. Control samples and serum standards with concentrations that ranged from 6.25 to 200 μg l^−1^ were included in each run. The limit of detection (lowest standard) was 6.25 μg l^−1^. All sCD163 measurements were performed at the Department of Clinical Biochemistry, Aarhus University Hospital.

### Separation of immune cell subsets and stimulation studies

EDTA tubes of blood were collected from healthy participants, and separation of PBMCs on Ficoll-Paque was performed. A number of cell subsets were isolated from the PBMCs, including monocytes, plasmacytoid dendritic cells, natural killer cells, B cells, T cells and neutrophils by EasySep isolation kits in accordance with the manufacturer's instructions. moDCs were generated from CD14-positive separated cells by a 6-day culture in granulocyte–macrophage colony-stimulating factor (GM-CSF; 67 ng ml^−1^; eBioscience, San Diego, CA, USA) and recombinant human IL-4 (17 ng ml^−1^). Macrophages were generated from monocytes by stimulation with GM-CSF or macrophage colony-stimulating factor (M-CSF) for 6 days in culture. Purity was checked by flow cytometry after cell separation and was >90% in each case.

### Real-time PCR

RNA was extracted from liver tissue of 94 well-characterized patients with CHC who underwent liver biopsy at Westmead Hospital and had stored liver tissue or serum, with no additional risk factors for liver steatosis or fibrosis; that is, diabetes, obesity (BMI>30 kg m^−2^), significant alcohol intake (>20 g per day), dyslipidaemia or use of cannabis and 28 sex- and age-matched adult healthy controls with benign liver tumours for whom all causes of liver disease were excluded, from PAXgene blood RNA tubes (*n*=75 CHC) using the RNeasy kit (Qiagen) according to the manufacturer's instructions. RNA quality and concentration was assessed using the Agilent 2100 Bioanalyser (Agilent, Waldbronn, Germany). cDNA was prepared using qscript (Quanta Biosciences, Gaithersburg, MD, USA) in a Mastercycler gradient 5331 (Eppendorf AG, Hamburg, Germany). Gene expression for MBOAT7, TNF-α, IL-1b, IL-6, CCL-19 and CCL-21 was measured by qPCR. GAPDH was used as the house-keeping gene. Expression was measured using CT values, normalized to that of GAPDH (ΔCT=CT (GAPDH) −CT (target) and then expressed as 2^ΔCT^. All amplifications were done in duplicate. qPCR primer sequences are listed in Supplementary Table 10.

### Immunohistochemical staining

Immunostaining for MBOAT7 was performed on liver biopsies of CHC patients. Formalin-fixed, paraffin-embedded 4 μm sections were stained using a Ventana Benchmark Immunostainer (Ventana Medical Systems, Inc, Arizona, USA). Slides were incubated with a dilution of 1:10 of rabbit polyclonal antibody (Ab) specific for Human MBOAT7 (SIGMA ALDRICH). Negative controls where the primary antibody was excluded confirmed the specificity of immunostaining. Detection was performed using Ventana's Ultra View DAB kit (Roche/Ventana 05269806001). The pathologist (D.M.) evaluated the MBOAT7 immunostaining semi-quantitatively in a blinded fashion regarding any of the histological and clinical characteristics of the patients. The extent of staining was scored according to its amount and intensity using a 4-point scoring system as follows: 0=no staining; 1=positive staining in <33% of cells; 2=33–66% of positive cells; and 3=positive staining in more than 66% of cells.

### Bioinformatic analysis of the rs641738 SNP and functional prediction

To identify the potential functional effects of rs641738 on MBOAT7 expression in blood, data sets that have evaluated constitutive RNA expression by mapping eQTLs in peripheral blood samples from 8,086 individuals was interrogated^28^. With the use of the ENCODE data (http://www.genome.gov/10005107)^29^, we conducted a bioinformatics analysis to predict the function of *MBOAT7*rs641738. The ENCODE data provide a multitude of experimental data suitable to annotate regulatory variants outside of protein-coding regions, and this was achieved by the Regulome DB (http://www.regulomedb.org)^30^, a fairly comprehensive variant annotation tool that makes use of functional sources. We also predicted whether the rs641738 is located in the microRNA-binding sites using an online-tool mirSNP (http://202.38.126.151/hmdd/mirsnp/search/)^31^.

### Statistical analysis

Statistical analyses were performed using the statistical software package SPSS for Windows, version 21(SPSS, Chicago, IL). Results are expressed as mean±s.d., median and interquartile range or number (percentage) of patients. The Student's *t*-test or non-parametric, that is, Wilcoxon–Mann–Whitney *U*-test or Kruskal–Wallis tests were used to compare quantitative data, as appropriate. *χ*^2^-test and Fisher-exact tests were used for comparison of frequency data and to evaluate the relationships between groups and the Cochran–Armitage test for trend. All tests were two-tailed and *P* values <0.05 were considered significant.

Multivariable regression modelling with backward elimination was undertaken to test independent associations of the *MBOAT7* rs641738 variant with the following outcome variables: (a) steatosis; (b) inflammation; and (c) fibrosis using ordinal regression models for ordinal traits (severity of steatosis, necroinflammatory activity and stage of fibrosis), and logistic regression models to examine binary traits; steatosis dichotomized as absent (S0) versus steatosis of any degree (≥S1), and in another analysis, as absent/mild (S0–S1) or moderate/severe (S2–S3); necroinflammation was dichotomized as absent/mild (METAVIR score A0–A1) or moderate/severe (METAVIR score A2–A3), and fibrosis as no fibrosis (F0) and fibrosis (F1–F4), absent/mild (METAVIR score F0–F1) or significant (METAVIR score F2–F4), none/moderate fibrosis (F0–F2) or severe fibrosis (F3–F4) and no cirrhosis (F0–F3) and cirrhosis (F4). Final models were assessed using the Hosmer–Lemeshow goodness of fit and Pearson goodness-of-fit tests. Analyses were adjusted for biologically relevant covariates and potential confounders associated with the risk of liver disease progression (age, gender, BMI, HOMA-IR, recruitment centre, alcohol consumption, viral load, *PNPLA3* rs738409, *TM6SF2* rs58542926 and *IFNL* rs12979860 genotype). HCV-RNA levels were log-transformed before entry into the model. Results are expressed as beta *β*±s.e. or OR and 95% CI. The OR estimates the relative change in the rate of the outcome (for example, significant fibrosis) per unit increase in the explanatory variable. Interactions among genes were tested by adding the corresponding gene X gene (GXG) interaction terms to the multivariable-adjusted regression models.

FPR was determined by calculating the ratio between the fibrosis stage and the estimated disease duration (in years). For FPR analysis, patients were stratified into two groups of stage-constant FPR according to the median rate (0.076 fibrosis units per year), which was used as a cutoff. We then used Cox regression analysis to model the time taken for any fibrosis (≥F1) to develop. For this, after checking the normality of the quantified variables, appropriate logarithmic transformations were made. We considered estimated age at infection as the starting point and the liver biopsy showing any fibrosis (failure time) or the liver biopsy showing an absence of any fibrosis in the absence of treatment (censored time) as the end point. A Cox proportional-hazards regression model was fitted, and the covariates were considered significant if *P*<0.05.Multivariate adjusted analyses were used, with age, gender, BMI, *PNPLA3* rs738409, *TM6SF2* rs58542926 and *IFNL* rs12979860 genotype as covariates.

Multivariable regression modelling with backward elimination was undertaken to test independent associations with hepatic MBOAT7 mRNA expression. Analysis was adjusted for age, sex, BMI, ALT and severity of liver disease. Final models were assessed using the Hosmer–Lemeshow goodness of fit and Pearson goodness-of-fit tests. Similarly, another model adjusting for severity of liver disease and leukocyte count was applied to assess the independent association of rs641738 with serum sCD163 levels.

We examined five potential genetic models that might explain the effect of rs641738 on liver histology outcome: co-dominant; dominant; recessive; over dominant; and additive. We investigated which model was the most appropriate by calculating the AIC values^32^. The lowest AIC is indicative of the best fit. The strength of the association between rs641738 and moderate/severe inflammation under each model was expressed by ORs and their corresponding 95% CIs. Using the CaTS power calculator for genetic association studies^33^, and assuming rs641738 MAF of 0.45, our cohort had 98% power for the dominant genetic model for the recessive allele and 96% power for the recessive one for liver histology outcomes (steatosis, inflammation and fibrosis).

### Data availability

The data that support the findings of this study are available from the corresponding author on a reasonable request.

## Additional information

**How to cite this article:** Thabet, K. *et al.* MBOAT7 rs641738 increases risk of liver inflammation and transition to fibrosis in chronic hepatitis C. *Nat. Commun.* 7:12757 doi: 10.1038/ncomms12757 (2016).

## Supplementary Material

Supplementary Information Supplementary Figures 1-2, Supplementary Tables 1-10 and Supplementary References
