# Supplementary material for: MBOAT7 rs641738 increases risk of liver inflammation and transition to fibrosis in chronic hepatitis C
Source: Nat Commun. 2016 Sep 15;7:12757. doi: 10.1038/ncomms12757 (PMC5027609; doi:10.1038/ncomms12757)
Supplement: Supplementary Information — Supplementary Figures 1-2, Supplementary Tables 1-10 and Supplementary References [file ncomms12757-s1.pdf]

SUPPLEMENTARY INFORMATION

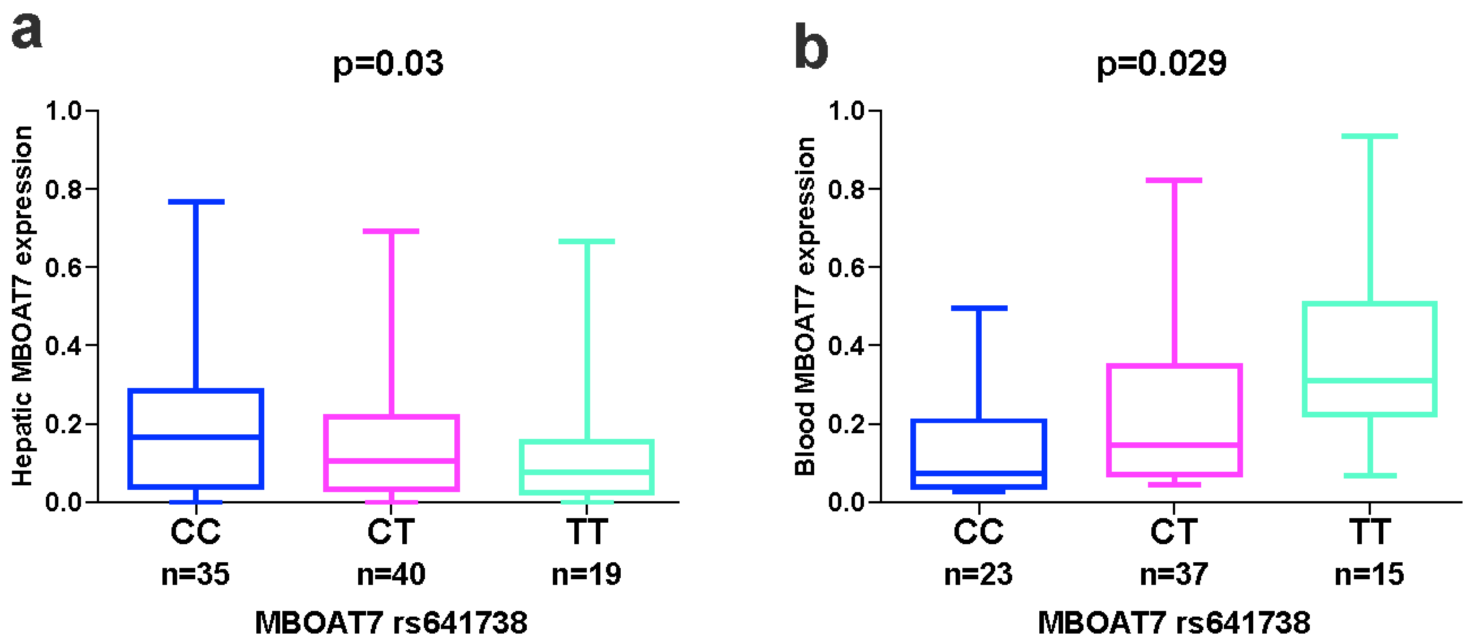

Supplementary Figure 1:Association between MBOAT7 genotype rs641738 and hepatic (A) and blood (B) MBOAT7 mRNA levels. The x axis shows the genotypes at rs641738using the additive model of inheritance, and the y axis shows the MBOAT7 expression level determined relative to GAPDH by qRT-PCR. The number of independent samples tested in each group is shown in parentheses. Each group is shown as a box plot and the median values are shown as thick dark horizontal lines. The box covers the twenty-fifth to seventy-fifth percentiles.P-value for trend was calculated using the Cochran–Armitage test for trend. We plotted the box plots using Graphpad prism 5.

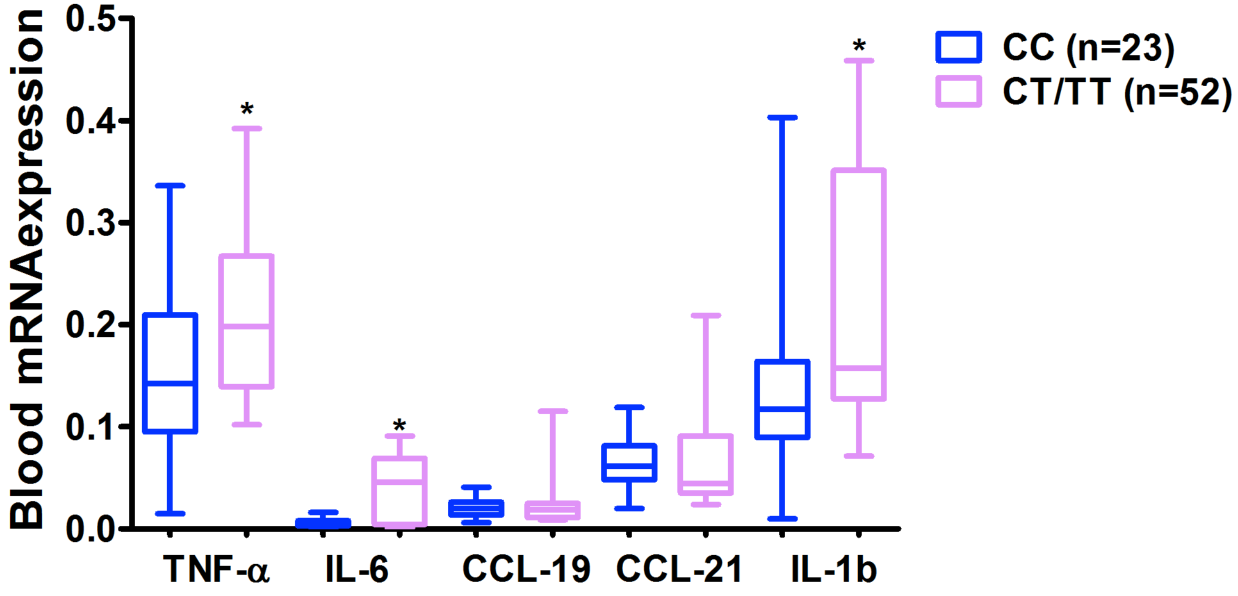

Supplementary Figure 2:Association between MBOAT7 genotype rs641738 and blood mRNA expression of TNF-α, IL-6, CCL-19,CCL-21 and IL-1b. The x axis shows the genotype at rs641738using the dominant model of inheritance, and the y axis shows inflammatory cytokine blood expression level relative to GAPDH by qRT-PCR. The number of independent samples tested in each group is shown in parentheses. Each group is shown as a box plot and the median values are shown as thick dark horizontal lines. The box covers the twenty-fifth to seventy-fifth percentiles.We tested the difference in the median values among genotypes using the two-tailed Mann–Whitney test.* $P < 0.05$ between patients with CC and CT/TT genotype. We plotted the box plots using Graphpad prism 5.

Supplementary Table 1: Demographic and clinical characteristics of the discovery (n=931), validation (n=775) and overall cohort (n = 1706) of patient with CHC.

Variables	Discovery Cohort(n=931)	Validation Cohort (n=775)	Overall (n=1706)	*P-value Discovery vs. Validation*
**Age (Years)**	44 (38-50)	46 (37-55)	44.9 (38-52)	0.0001
**Male (%)**	630 (68)	471 (60.7)	1101 (64.5)	0.003
**BMI(Kg/m^2)**	25.6 (23.5-28.9)	25.7 (23.4-28)	26 (23.5-29)	0.1
**T2DM (%)**	66 (0.07)	54 (0.069)	120 (0.07)	0.9
**ALT (IU/L)**	81 (49-142)	69 (42-111)	74 (46-128)	0.0001
**AST (IU/L)**	58 (40-94)	48 (34-72)	53 (37-86)	0.0001
**GGT (IU/L)**	57 (30-104)	43 (24-87)	50 (26-92)	0.0001
**Platelet (x10^9/L)**	210 (170-257)	208 (163-252)	210 (167-255)	0.7
**Leukocytes (x10^9/L)**	6.8 (5.6-8.4)	6.6 (5.4-8)	6.7 (5.5-8.1)	0.1
**Cholesterol (mmol/L)**	4.5 (3.8-5.1)	4.4 (3.8-5)	4.4 (3.8-5)	0.5
**Triglycerides (mmol/L)**	0.97 (0.75-1.33)	0.96 (0.75-1.35)	0.96 (0.75-1.34)	0.8
**LDL-C (mmol/L)**	2.5 (2.1.-3.1)	2.6 (2.2-3.3)	2.6 (2.1-3.2)	0.097
**HDL-C (mmol/L)**	1.3 (1.1-1.6)	1.3 (1.1-.58)	1.3 (1.1-1.6)	0.8
**Blood glucose (mmol/L)**	5.1 (4.7-5.5)	5.05 (4.7-5.5)	5.1 (4.7-5.5)	0.051
**HOMA-IR**	2.3 (1.4-3.9)	2.04 (1.41-3.39)	2.18 (1.4-3.8)	0.2
**HCV-RNA log$_{10}$**	5.92 (5.59-5.92)	5.84 (5.49-6.31)	5.9 (5.55-6.18)	0.2
**HCV-genotype 1, 2, 3, 4 (%)**	565 (60.7), 65 (7), 274 (29.4), 27 (2.9)	555 (71.6), 137 (17.7), 69 (8.9),14 (1.8)	1120 (65.7), 202 (11.8), 343 (20.1), 41 (2.4)	0.0001
**Alcohol history (%)**<br>None or less than 50 g/daily (%)<br>≥ 50 g/daily (%)	 773 (83)<br>158 (17)	 659 (85)<br>116 (15)	 1432 (84)<br>274 (16)	0.2

**Liver fibrosis (%)**				0.1
F0-F1	438 (47)	338 (43.6)	776 (45.5)	
F2-F4	493 (53)	437 (56.4)	930 (54.5)	
**Inflammation score (%)**				
A0-A1	504 (54)	440 (57)	944 (55)	
A2-A3	427 (46)	335 (43)	762 (45)	0.2
***IFNL*rs12979860 genotype (%)**				
CC	354 (38)	271 (35)	625 (36.6)	
CT	460(49.4)	385 (49.7)	845 (49.6)	0.1
TT	117 (12.6)	119 (15.3)	236 (13.8)	
***TM6SF2* rs58542926 genotype (%)**				
CC	810 (87)	689 (88.9)	1499 (87.9)	
CT	114 (12.2)	82 (10.6)	196 (11.5)	
TT	7 (0.8)	4 (0.5)	11 (0.6)	0.4
***PNPLA3* rs738409 genotype (%)**				
CC	513 (55.2)	430 (55.5)	943 (55.3)	
CG	352 (37.8)	287 (37)	639 (37.4)	0.9
GG	66 (7.1)	58 (7.5)	124 (7.3)	

Data are presented either median plus interquartile range (25th, 75th percentile) or number (percentage) of patients. HCV-RNA viral load levels were available for 1318 patients. Lipid profile data were available for 983 patients. BMI: body mass index; T2DM: type 2 diabetes mellitus, HOMA-IR, homeostatic model assessment of insulin resistance (HOMA-IR)

Supplementary Table 2: Distribution of *MBOAT7 rs641738* genotypes and Hardy-Weinberg equilibrium.

MBOAT7 rs641738	**CHC (European population) (n=1706)**	**Healthy controls (European population) (n=270)**
CC	531 (31)	84 (31)
CT	822 (48)	127 (47)
TT	353 (21)	59 (22)

p =0.1 for both CHC and healthy controls cohorts. P values were calculated by chi square test, p>0.05 indicates no deviation from Hardy-Weinberg equilibrium

Supplementary Table 3. Odds ratio, Akaike's Information Criterion (AIC) values for various rs641738 genetic models of inheritance.

Genetic Model	OR (95% CI)	P-value*	AIC
**Additive model**			
**CC**	**Reference**	0.007	2341.7
**CT**	1.205 (1.01-1.51)		
**TT**	1.45 (1.15-1.84)		
**Dominant model (for minor allele)**			
**CC**	**Reference**	0.001	2339.8
**CT/TT**	1.40 (1.13-1.69)		
**Recessive model**			
**CC/CT**	**Reference**	0.004	2340.8
**TT**	1.37 (1.12-1.73)		

OR, Odds ratio; AIC, Akaike information criterion. Lower AIC values indicate a better fit.

Supplementary Table 4: Characteristics of 1706 patients with CHC according to *MBOAT7* rs641738 genotype*

Variable	*MBOAT7* rs641738 Genotype		P-value
	CC (n=531)	CT/TT (n=1175)	
**Age at time of biopsy (yrs)**	44.5 (37.7-52.4)	45 (38-51.8)	0.9
**Male Gender (%)**	349 (65.7)	752 (63.9)	0.5
**HCV genotype 3 (%)**	109 (21)	234 (20)	0.1
**HCV-RNA $\log_{10}$**	5.9 (5.4-6.1)	5.9 (5.6-6.2)	0.1
**Body Mass Index (Kg/m^2)**	26 (23.7-29.2)	26 (23.3-29)	0.4
**ALT (IU/L)**	75 (47-125)	74 (46-132)	0.7
**AST (IU/L)**	56 (38-81)	52 (37-87)	0.5
**GGT (IU/L)**	48 (26-90)	51 (27-92)	0.4
**Alkaline phosphatase(IU/L)**	77 (64-97)	80 (64-108)	0.1
**Platelet (x10^9/L)**	211 (167-249)	210 (167-258)	0.7
**Leukocytes (x10^9/L)**	6.5 (5.4-7.7)	6.9 (5.5-8.4)	0.01
**Cholesterol (mmol/L)**	4.4 (3.8-5)	4.4 (3.8-5.1)	0.4
**Triglycerides (mmol/L)**	0.98 (0.75-1.4)	0.96 (0.75-1.3)	0.6
**LDL-C (mmol/L)**	2.6 (2.1-3.1)	2.6 (2.1-3.2)	0.4
**HDL-C (mmol/L)**	1.3 (1.1-1.6)	1.3 (1.07-1.6)	0.5
**Blood glucose (mmol/L)**	5.1 (4.7-5.6)	5.1 (4.7-5.5)	0.1
**HOMA-IR**	2.4 (1.4-3.7)	2.1 (1.4-3.8)	0.4

Data are presented either median plus interquartile range (25th, 75th percentile) or number (percentage) of patients. HCV-RNA viral load levels were available for 1318 patients. Lipid profile data were available for 983 patients.

Supplementary Table 5: Independent predictors of moderate/severe steatosis (≥S2), severe necroinflammation (≥A2) and presence of fibrosis (≥F1)

	Degree of steatosis (≥S2)			Inflammation (≥ A2)			Anyfibrosis (≥ F1)		
	OR	95% CI	*P* value	OR	95% CI	*P* value	OR	95% CI	*P* value
**Age, years**	1.01	0.998-1.02	0.09	1.02	1.009-1.04	0.003	1.03	1.01-1.05	0.002
**Gender, female**	0.97	0.62-1.5	0.9	0.81	0.573-1.15	0.2	0.887	0.636-1.238	0.4
**HOMA-IR**	1.05	1.01-1.08	0.006	1.11	1.04-1.19	0.001	1.1	0.99-1.22	0.06
**BMI, Kg/m^2**	1.07	1.01-1.14	0.01	1.03	0.99-1.07	0.05	0.998	0.994-1.05	0.9
**Alcohol (≥ 50 g/daily)**	1.02	1-1.04	0.049	1.24	0.95-1.62	0.1	1.29	0.91-1.82	0.1
**HCV-RNA log_{10}**	1.06	0.84-1.33	0.6	1.29	0.98-1.73	0.09	1.05	0.81-1.37	0.6
***PNPLA3* rs738409**	2.16	1.41-3.31	0.0001	1.17	0.80-1.72	0.4	1.1	0.67-1.77	0.6
***TM6SF2* rs58542926**	1.13	1.01-1.24	0.024	1.03	0.71-1.5	0.8	1.09	0.6-1.97	0.7
***IFNL* rs12979860**	1.15	0.82-1.61	0.4	2.21	1.71-2.86	0.0001	1.24	1.04-1.89	0.03
***MBOAT7* genotype**	0.99	0.74–1.33	0.9	1.44	1.14-1.72	0.001	1.69	1.11-3.57	0.02

Multiple Logistic regression models were used to test the association MBOAT7 with liver histology outcomes (steatosis, inflammation andfibrosis). In addition to predictors shown here, models were adjusted for recruitment centre. Genetic analyses were undertaken using a dominant model, except for PNPLA3, where an additive dominant model was used. A dominant model was applied for TM6SF2 (due to the low minor allele frequency) and for IFNL and MBOAT7 as they best fitted the data with the smallest Akaike Information Criterion (AIC) values. The reference group was defined by the absence of moderate or severe steatosis (S0-S1), absent/mild (METAVIR score A0-A1) inflammation,and no fibrosis (F0).

Supplementary Table 6: Demographic and clinical characteristics of the sub-cohort (n = 1080) of patients with CHC and a known estimated duration of infection compared to the overall cohort (n=1706).

Variables	FPR sub-cohort* (n=1080)	Overall cohort (n=1706)	*P-value FPR vs. overall cohort*
**Age**	44.8 (38-51)	44.9 (38-52)	0.8
**Male (%)**	716 (66.3)	1101 (64.5)	0.3
**BMI(Kg/m^2)**	26 (23.5-29)	26 (23.5-29)	0.9
**ALT (IU/L)**	79 (48-137)	74 (46-128)	0.3
**AST (IU/L)**	57 (39-90)	53 (37-86)	0.5
**GGT (IU/L)**	53 (28.8-99)	50 (26-92)	0.5
**Platelet (x109/L)**	217 (174-263)	210 (167-255)	0.4
**HCV-RNA log$_{10}$**	5.91 (5.55-6.03)	5.9 (5.55-6.18)	0.8
**HCV-genotype 3 (%)**	220 (20.2)	343 (20.1)	0.8
**Liver fibrosis**			
**None/mild**	504 (46.7)	776 (45.5)	0.5
**Moderate/severe**	576 (53.3)	930 (54.5)	
**Inflammation score**			
**None/mild**	595 (55.1)	944 (55)	0.9
**Moderate/severe**	485 (44.9)	762 (45)	
**Steatosis degree**			
**None/mild (%)**	894 (82.8)	1415 (83)	0.9
**Moderate severe (%)**	186 (17.2)	291 (17)	

Fibrosis progression rate (FPR) was calculated by taking the ratio between the fibrosis stage and the estimated disease duration (in years). Data are presented either median plus interquartile range (25th, 75th percentile) or number (percentage) of patients.

Supplementary Table 7: *MBOAT7* rs641738 genotype distribution in HCV-related HCC (n=75)

***MBOAT7* rs641738**	**HCC cohort (n=75)**
CC	24 (0.32)
CT	35 (0.47)
TT	16 (0.21)

p =0.6. P values were calculated by chi square test, p>0.05 indicates no deviation from Hardy-Weinberg equilibrium.The MBOAT7 rs641738 allele and genotype frequencies were compared in 75 Caucasian patients with HCV-related HCC to the entire CHC cohort described above (n=1,706). rs641738 MAF in the HCC cohort was 0.44, similar to the CHC cohort.

Supplementary Table 8: Clinical characteristics of sub-cohorts of CHC subjects from whom MBOAT7 hepatic mRNA, blood MRNA and inflammatory markers was measured.

Variable	Hepatic mRNA (n=94)	Blood mRNA (n=75)	ELISA (n=95)
**Age at time of biopsy (yrs)**	44.7 (38-52)	45 (37-54)	43.8 (37-49)
**Male Gender (%)**	63 (67)	49 (65)	60 (63)
**Body Mass Index (Kg/m^2)**	26.8 (24-30)	25.9 (23.9-29.1)	25.7 (23.4-28.5)
**HCV-RNA log$_{10}$**	5.92 (5.45-6.22)	5.9 (5.26-6.49)	5.92 (5.55-6.12)
**ALT (IU/L)**	74 (45-137)	84 (52-144)	80 (50-137)
**AST (IU/L)**	52 (41-100)	54 (37-102)	50 (38-90)
**Platelet (x10^9/L)**	213 (192-269)	197 (179-245)	206 (178-257)
**Leukocytes (x10^9/L)**	6.9 (5.7-8.4)	6.35 (5.3-8.1)	6.78 (5.6-8.1)
**Cholesterol (mmol/L)**	4.5 (3.9-5.1)	4.5 (3.8-4.8)	4.3 (3.6-4.85)
**Triglycerides (mmol/L)**	1.08 (0.78-1.48)	1.11 (0.91-1.6)	0.97 (0.8-1.11)
**LDL-C (mmol/L)**	2.5 (2-3.1)	2.65 (2.2-3.15)	2.6 (2.1-3.2)
**HDL-C (mmol/L)**	1.35 (1-1.67)	1.16 (0.93-1.37)	1.3 (1.1-1.5)
**Blood glucose (mmol/L)**	5.2 (4.8-5.6)	5.3 (5-5.9)	5.2 (4.9-5.6)
**HOMA-IR**	2.23 (1.46-4.02)	2.26 (1.56-3.73)	1.94 (1.3-2.6)

Data are presented either median plus interquartile range (25th, 75th percentile) or number (percentage) of patients.

Supplementary Table 9: Bioinformatics screen of rs641738 functional effects using the Regulome DB tools

SNP	Regulome DB score	Motifs		Protein binding		Chromatin structure		Histone modifications	
		**Method**	**Motif**	**Method**	**Bound protein**	**Method**	**Chromatin structure**	**Method**	**Histone Mark**
**rs641738**	**1b**	PWM Footprinting	1 multiple	ChIP-seq	multiple	DNase-seq	Multiple	ChromHMM	Multiple

When the number of predicted transcription factor binding motifs (or protein binding sequences, chromatin structures, histone marks) is more than 10, we have indicated it as "multiple". The methods for the bioinformatics screen on ENCODE data have been described previously, and can be found in Boyle et al (1). Regulomedb scores are from 1a to 6 and the lower the score, the more likely that the SNP affects transcription factor binding and is linked to the expression of a gene target.

Supplementary Table 10: Primer Sequences Used for qRT-PCR.

Gene	Primer	Sequence 5` to 3`
MBOAT7	forward	CCTGCTCTCCTCTCACCTCT
	reverse	AATCCAGGCCACGTAGAAGC
TNF-α	forward	TCTCTAATCAGCCCTCTGGCCCAGG
	reverse	TACAACATGGGCTACAGGCTTGTCAC
IL-1β	forward	ACAGATGAAGTGCTCCTTCCA
	reverse	GTCGGAGATTCGTAGCTGGAT
IL-6	forward	AAATGCCAGCCTGCTGACGAAC
	reverse	AACAACAATCTGAAGTGCCCATGCTAC
CCL19	forward	GCCTGCTGGTTCTCTGGAC
	reverse	GGATGGGTTTCTGGGTCAC
CCL21	forward	CCAAAGGCTGCAAGAGGA
	reverse	CTTCAAGCGCTGGTGAGG

Supplementary Reference

1. Boyle AP, *et al*. Annotation of functional variation in personal genomes using RegulomeDB. *Genome Res*; **22**:1790–1797 (2012).
